# Host-microbiome interactions: the aryl hydrocarbon receptor and the central nervous system

**DOI:** 10.1007/s00109-016-1486-0

**Published:** 2016-11-17

**Authors:** Hae Ung Lee, Zachary E McPherson, Bryan Tan, Agata Korecka, Sven Pettersson

**Affiliations:** 1 The LKC School of Medicine, Nanyang Technological University, Singapore, Singapore; 2 The School of Medicine and Public Health, University of Newcastle, Newcastle, Australia; 3 The School of Medicine, Imperial College, London, UK; 4 Department of Microbiology, Cell and Tumor Biology, Karolinska Institutet, Solna, Sweden

**Keywords:** Host-microbiome interactions, Neurodevelopment, Neurodegeneration, Aryl hydrocarbon receptor

## Abstract

The microbiome located within a given host and its organs forms a holobiont, an intimate functional entity with evolutionarily designed interactions to support nutritional intake and reproduction. Thus, all organs in a holobiont respond to changes within the microbiome. The development and function of the central nervous system and its homeostatic mechanisms are no exception and are also subject to regulation by the gut microbiome. In order for the holobiont to function effectively, the microbiome and host must communicate. The aryl hydrocarbon receptor is an evolutionarily conserved receptor recognizing environmental compounds, including a number of ligands produced directly and indirectly by the microbiome. This review focuses on the microbiome-gut-brain axis in regard to the aryl hydrocarbon receptor signaling pathway and its impact on underlying mechanisms in neurodegeneration.

## Introduction

The alimentary tract contains trillions of microbes with overlapping biological and biochemical needs due to coevolutionary mechanisms, collectively termed the gut microbiome. Though researchers have shown that the gut microbiome impacts virtually all aspects of host function, the mechanisms and signaling pathways by which the gut microbiota communicates with its host are still unknown.

Bacteria and archaea, two of the predominant kingdoms within the microbiome, were the dominant forms of life on Earth for approximately three billion years prior to the evolution of the animal kingdom [1, 2]. Current understanding increasingly considers the host and its microbiome as a working functional unit known as the holobiont. Environmental changes affect both the host and its microbiome. The last decade of genome-wide association studies has ignored the microbiome and, consequently, missed the response elicited within it. In the last 20 years, germ-free (GF) mice, mice that are raised without exposure to any microbes, have been used to address the holobiont concept using a systems biology approach [3]. A prerequisite for a holobiont to function is the ability of the host and microbiome to communicate, to maintain homeostasis, and to act correspondingly when exposed to assaults. We postulate that many of the ligands and receptors identified and used for host-microbiome interactions are evolutionary. This review focuses on the well-described xenobiotic aryl hydrocarbon receptor (AHR) as one possible evolutionarily conserved signaling pathway that contributes to host-microbiome homeostasis within the holobiont.

### The aryl hydrocarbon receptor

The AHR is a cytoplasmic ligand-induced receptor originally discovered as a xenobiotic sensor mediating the toxicity of 2,3,7,8-tetrachlorodibenzo-p-dioxin (TCDD), also known as dioxin [4–7]. The metabolism of xenobiotic compounds is initiated by activation of the AHR, which then translocates to the nucleus, where it acts as a transcription factor for specific target genes, such as cytochrome P450 1A1 and cytochrome P450 1B1 [4, 5, 8–12]. However, invertebrates do not have a toxic response to dioxin, and none of the currently known invertebrate AHR orthologues, including spineless in *Drosophila*, have dioxin binding capacity, which suggests that the ancestral role of the AHR is not specifically toxin response [13, 14]. Furthermore, physiological roles of the AHR in responses to endogenous ligands have been reported in cell cycle regulation, cell differentiation, and immune responses [11, 15–18]. A number of endogenous AHR ligands have been suggested through in silico research and biological testing, including tryptophan metabolites [5, 11, 19]. Recently, our group discovered that AHR expression is attenuated in GF mice [20]. This finding suggests that the AHR acts as a mediator in communication between the host and gut microbiota.

### Function of the aryl hydrocarbon receptor in host-environment interactions

Dioxin-activated AHR attenuates lipid metabolism via negative regulation of peroxisome proliferator-activated receptor (PPAR) [21]. Dysregulation of lipid metabolism leading to hepatic steatosis and insulin resistance suggests that the AHR plays an important role in integrating exogenous and endogenous influences in lipid and energy metabolism [22, 23]. Findings from AHR-deficient mice show that, like GF mice [24, 25], they are protected from high fat diet-induced obesity, hepatic steatosis, and insulin resistance [26].

Recently, fibroblast growth factor 21 (FGF21) was reported to be a novel target gene of the AHR. FGF21 increases lipid oxidation and ketogenesis but decreases gluconeogenesis at the gene expression level [27, 28]. As an insulin sensitizer, FGF21 boosts the metabolic benefits such as improved blood glucose levels due to increased glucose uptake in adipocytes, reduced body weight due to increased energy expenditure, and improved blood lipid profiles due to hepatic sequestration of lipid droplets [29–31]. TCDD-induced AHR activation has been shown to increase FGF21 messenger RNA (mRNA) in both a dose- and time-dependent manner in mouse liver [22, 23]. In addition, drug-induced overexpression of human AHR in mice induces the activation of FGF21 which may then result in decreased insulin resistance [32]. The opposite effects were observed with the downregulation of FGF21—insulin insensitivity, deranged lipid profile, and liver inflammation—and can be associated with the attenuation of hepatic lipid accumulation and increased transfer of fats out of the liver in hepatocyte-targeted AHR knockout (KO) [22]*.*

Recent work from our lab linked the mechanism of microbiota and host communication through an AHR-dependent mechanism. We demonstrated that the AHR is differentially expressed in GF mice. Similarly, our AHR-KO study showed that AHR regulates a set of metabolic genes in the liver, including CD36 (involved in fatty acid uptake) and Hmgcs2 (an enzyme involved in ketone body regulation) [20]. Similar to fast-induced adipose factor-KO mice [25], AHR-KO mice gain weight as expected but do not develop insulin resistance [20], suggesting that AHR could be the upstream link between microbiota-mediated signals and the host [20].

*S*everal reports have associated AHR function with the regulation of the immune system. TCDD treatment has shown that AHR has the capacity to mediate the differentiation and/or function of T cells, macrophages, and dendritic cells [10, 12, 15, 33–37]. The activation of AHR by TCDD [38–40] and the ablation of AHR in KO animals [41] have implicated this receptor in viral immunity. We also recently reported that ablating the AHR in CD11c^+^ cells perturbs the development of the intestinal epithelium and intestinal immunity [42]. Depending on the presence of specific ligands, AHR activation has also been shown to suppress or exacerbate responses in experimental autoimmune disease models. For example, TCDD and 2-(1′H-indole-3′-carbonyl)-thiazole-4-carboxylic acid methyl ester (ITE) can suppress experimental autoimmune encephalomyelitis (EAE), a model of multiple sclerosis (MS) [35], whereas the activation of AHR by ligands such as 6-formylindolo[3,2-b]carbazole (FICZ) exacerbates the development of EAE [16, 35, 37, 43]. In addition, the affinity of AHR for ligands (TCDD, high affinity; FICZ, low affinity) influenced the amount of IL-17 and IL-22 protein secreted by Th17 cells [44]. These findings indicate that various ligands for AHR may have different effects on host development.

### Natural ligands for the aryl hydrocarbon receptor

**Fig. 1:**
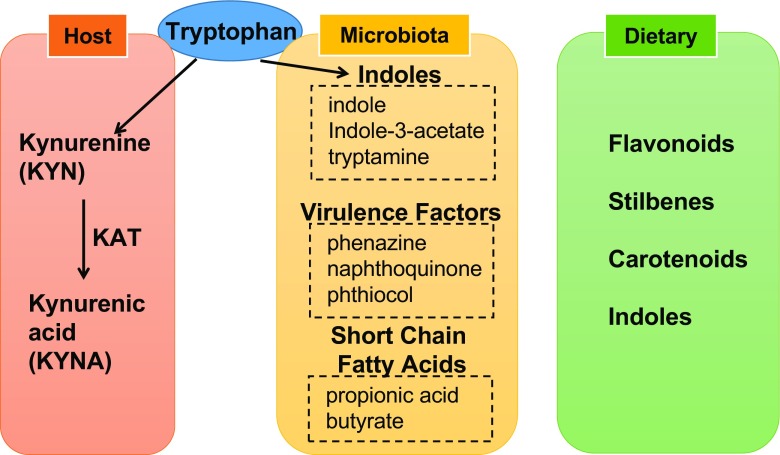
Natural ligands for aryl hydrocarbon receptor. Kynurenine (*KYN*) is converted from tryptophan in host metabolism. Kynurenic acid (*KYNA*) is also an AHR ligand, converted from KYN by kynurenine aminotransferase (*KAT*). There are three groups for microbiota-mediated AHR ligands: (1) tryptophan metabolites derived by microbiota, (2) bacterial virulence factors, and (3) short chain fatty acids. Short chain fatty acids are not direct ligands for AHR, but those facilitate AHR effects. Flavonoids, stilbenes, carotenoids, and indoles from plants are dietary ligands for AHR

Though most research on AHR has focused on man-made high affinity binding ligands and chemical pollutants, recent research has implicated important roles for an array of low affinity natural ligands produced, metabolized, or influenced by the gut microbiota. Natural ligands for AHR can be divided into three groups: host mediated, microbiota mediated, and dietary (Fig. 1).

The essential amino acid tryptophan is the major source for both host-mediated and microbiome-mediated AHR ligands. Kynurenine (KYN) is converted from tryptophan by tryptophan 2,3-dioxygenase (TDO) or indoleamine 2,3-dioxygenase (IDO) and is an important AHR ligand [44]. Kynurenic acid (KYNA) is converted from KYN by kynurenine aminotransferase and also an important ligand [45]. Our research has shown that the microbiota regulates the expression of IDO in the liver, and although IDO may play a more important role in KYN metabolism in extrahepatic tissue [46]. These results indicate a need to analyze the role of the microbiota in KYN metabolism [20].

**Fig. 2:**
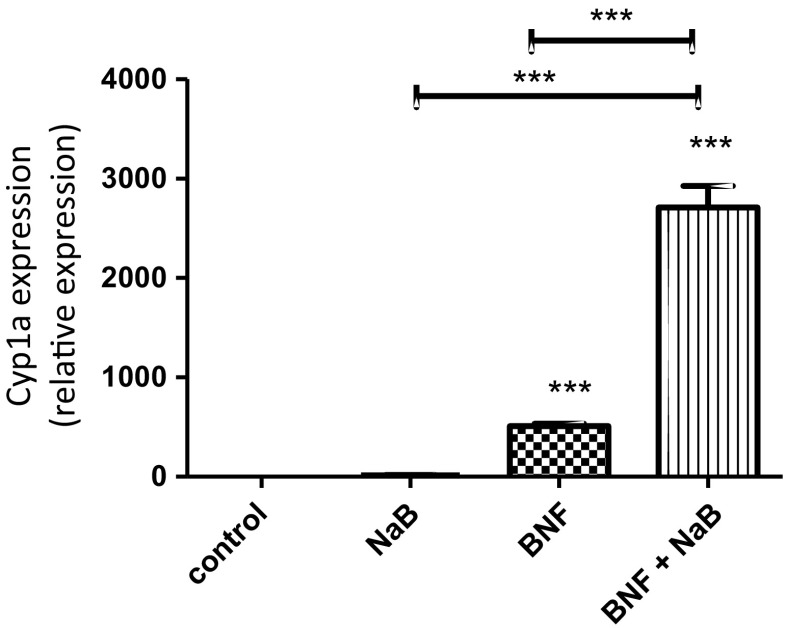
Sodium butyrate (*NaB*) increase the activity of the aryl hydrocarbon receptor (*AHR*). HepG2 cells were cultured for 24 h with normal media or media containing NaB, beta-naphthoflavone (*BNF*), a natural agonist for AHR or NaB and BNF. While NaB not having a direct ligand effect on AHR demonstrated a significant synergistic effect to increase the activation of cyp1a by BNF

Gut microbiota also converts tryptophan to indole, indole-3-acetate, and tryptamine, which have been identified in mouse and human intestine and work as AHR agonists and antagonists [47, 48]. Microbial pigment virulence factors, namely the phenazines from microbes such as *Pseudomonas aeruginosa* and the naphthoquinone phthiocol from *Mycobacterium tuberculosis*, act as microbiota-mediated AHR ligands. Upon ligand binding, AHR activation leads to virulence factor degradation and regulates cytokine and chemokine production [49]. Short chain fatty acids, such as propionic acid and butyrate, from the microbiome are not direct ligands for AHR, but our recent data suggest that they stabilize AHR, increasing its activity in the presence of true ligands (Fig. 2).

The majority of dietary AHR ligands are produced by plants. Plant-derived compounds that act as ligands for AHR include flavonoids, stilbenes, carotenoids, and some indoles. Indole-3-carbiol (I3C) is an indole compound found in cruciferous vegetables that is converted to higher affinity AHR ligands, such as indolo-[3,2-b]-carbazole and 3,3′-diindolymethane in the acidic environment of the stomach [50].

### Microbiome and neurodevelopment

An important aspect of the functionality of the holobiont is the ability of each component to shape the behaviors of the others. Simply put, it behooves the microbiota to encourage certain “healthy” behaviors in the host. Although the effects of the microbiota are important in maintaining metabolism and the immune system, it is logical to conclude that the microbiota, acting in the best interests of the whole holobiont, may play a pivotal role in supporting the development of the central nervous system (CNS).

#### The microbiota and neurodevelopment

Sudo et al. first demonstrated a possible link between the hypothalamic-pituitary-adrenal (HPA) axis and the gut microbiome [51]. Elevated adrenocorticotropic hormone and corticosterone levels were observed in GF mice compared to specific pathogen-free (SPF) mice in early life. They also demonstrated that brain-derived neurotrophic factor (BDNF) is significantly reduced in the hippocampus and cortex of GF mice [51]. Later studies confirmed regulation of steady state levels of BDNF by the microbiome [52, 53], which plays an important role in neuroplasticity, neuron differentiation, and the maintenance and protection of neurons under stress. Many of these groups have linked changes in brain biochemistry to altered behaviors in GF mice [51–53].

Recently, there have been reports that the microbiome plays an important role in the growth and function of CNS cell populations. Hippocampal neurogenesis was shown to be increased in GF mice [54], which also correlated to increased volume and abnormal neuronal morphology in the hippocampi of GF mice [55]. Similarly, there was increased amygdala volume in GF mice with concomitant neuronal morphology [55]. In contrast to this, hippocampal neurogenesis was shown to be decreased in mice treated with antibiotics [56]. Möhle et al. demonstrated that their model of antibiotic depletion leads to decreased hippocampal neurogenesis through modulation of the populations of specific immune cells [56]. Our group has also reported that the microbiome plays a key role in the maintenance of other synaptic proteins, including synaptophysin and PSD-95, both of which are reduced in the striatum of SPF mice, suggesting abnormally hyperactive synaptogenesis in the striatum of GF mice [53]. The microbiome has also been implicated in the functionality of glial cells. Our group has demonstrated that the microbiota is instrumental in the development of the blood-brain barrier (BBB) [57]. Finally, Hoban et al. demonstrated that in the absence of microbiota, there is increased myelination of neurons in the prefrontal cortex [58].

Though these observations are of interest, knowledge on the molecular mechanisms linking the microbiome to neurodevelopment remains limited.

#### The aryl hydrocarbon receptor in neurodevelopment

Reports on the AHR in neurodevelopment are very limited. However, the AHR appears to be vital in the maintenance of some key pathways in neurodevelopment in worms. In *Caenorhabditis elegans*, Huang et al. demonstrated a role for AHR in neural cell fate determination, particularly for GABAergic neurons [59]. AHR-1 is the AHR orthologue in *C*. *elegans*. In worms with AHR-1 mutations, two specific neurons out of the 302 total neurons have been reported to appear and act like a second pair of neurons that could be reprogrammed into the first pair of neurons by ectopic administration of AHR-1 [59]. In addition, Qin et al. found that AHR-1 is responsible for the development, orientation, and axonal migration of AHR-1-expressing neurons in *C*. *elegans* [60]. Taken together, these results demonstrate that AHR contributes to the cell fate determination of specific neuronal populations in worms, possibly through natural ligands and irrespective of dioxin exposure.

Dioxin toxicity studies have demonstrated that the AHR is likely to play a role in CNS development. In zebrafish, TCDD exposure was reported to reduce the total number of neurons by 30% [61]. In mice, dioxin toxicity studies have demonstrated a similar role for AHR in the embryonic differentiation of GABAergic neurons in the telencephalon [62] and the neurogenesis of cerebellar granule cells [63]. Importantly, due to the extraordinarily high binding affinity of dioxin for the AHR, emphatic conclusions regarding the physiological role of the AHR in normal development cannot be drawn from dioxin studies alone.

The AHR was also shown to play a crucial role in CNS development in studies more consistent with typical biology. The expression of a constitutively active AHR in mice retarded the development of interneurons in the olfactory bulb [64]. Furthermore, in mouse primary cortical neurons, AHR activation by FICZ was also shown to increase the expression of synaptophysin and SAP102, but not PSD95 [65]. In functional experiments, the AHR was shown to alter hippocampal neurogenesis and contextual fear memory in mice [66], as well as aggression behavior in *C. elegans* [67]. Latchney et al. demonstrated that adult AHR-KO mice and TCDD-exposed mice hippocampal-dependent memory impairment. AHR-deficient mice and TCDD-exposed mice also exhibited reduced cell proliferation, survival, and differentiation in the adult dentate gyrus [66]. The often conflicting data demonstrating both the KO and activation of AHR lead to similar outcomes, suggesting that the AHR plays a vital role in CNS homeostasis.

#### Autism spectrum disorder

Autism spectrum disorder (ASD) is a neurodevelopmental illness for which evidence supports a possible link between the maternal/early postnatal microbiome and dysfunctional neurodevelopmental programming. From a human health perspective, the association between the microbiome and neurodevelopment was highlighted by evidence that people suffering from ASD also frequently present with problems related to a dysfunctional bowel with aberrant intestinal barrier function [68]. Although the association remains controversial, a role for dysfunctional microbiome-gut-brain axis has gained further support from the recent demonstration of different microbiome composition in children with ASD compared to age-matched controls [69].

A recent study demonstrated that, in an animal model of ASD, correction of the microbiota with probiotic administration of *Bacteroides fragilis* corrected biochemical and behavioral abnormalities associated with ASD [70]. In this ASD mouse model, the key effector in the microbiota-gut-brain axis was the metabolome; a number of specific metabolites altered in the ASD mouse model were normalized by the treatment. Indolepyruvate, a microbially controlled molecule that is metabolized into an AHR agonist, was significantly regulated in the ASD model and by *B. fragilis* treatment [70]. This metabolite is an interesting corollary to indolyl-3-acryloylglycine, which has been shown to be elevated in the urine of humans with ASD [71].

Epidemiological studies of Vietnamese children exposed to TCDD in the prenatal and perinatal period have demonstrated increased neurodevelopmental defects and autistic traits in children with greater exposure to TCDD [72]. Prenatal and postnatal exposure to KYN in rats causes cognitive defects in adulthood [73]. Although Pocivavsek et al. did not identify a specific mechanism underlying the association between early life KYN exposure and cognitive deficits, they did note that the treatment led to 3.4- and 2.1-fold increases in KYNA levels in the brain at postnatal days 2 and 21, respectively [73]. Although Pocivavsek et al. noted the effects of KYNA as an antagonist of the α7 nicotinic acetylcholine receptor and the N-methyl-d-aspartate receptor [73], KYNA is also an AHR ligand with a stronger binding affinity for the AHR than KYN [45], potentially implicating AHR activity in the cognitive abnormalities observed in this model.

An animal model of ASD appearing to be caused, in part, by microbial metabolites that act on the AHR and epidemiological studies linking environmental exposure to AHR ligands to neurodevelopmental issues and strong associations between ASD and gastrointestinal pathology suggest that ASD is a systems biology problem within the holobiont. Therefore, the AHR signaling pathway and its microbially derived natural ligands are of great interest for further exploration of ASD and other neurodevelopmental disorders.

### Neurodegeneration

Neurodegeneration is regarded as a pathological process, whereby neuron loss is increased frequently in association with aging. The mechanisms underpinning neurodegeneration and neuron loss are poorly understood, but are assumed to be the result of a metabolic dysfunction, increased autophagy, and aberrant host immune system activity.

Irritable bowel syndrome (IBS), an illness associated with disruption of the microbiota, has been shown to be a risk factor for Parkinson’s disease [74] and both non-Alzheimer’s disease dementia and Alzheimer’s disease [75]. Our group also recently showed that IBS may precede glaucoma, a progressive neurodegeneration of the optic nerve, in two primarily Caucasian populations [76]. These results provide evidence that pathological mechanisms underlying IBS, including disruption of the microbiota, may have clinically relevant effects in neurodegenerative illnesses and alter homeostatic mechanisms in the CNS. Moreover, tryptophan metabolism by the microbiota has been suggested to play a role in IBS pathology through AHR-mediated pathways [77].

Parkinson’s disease, which has long been known to be associated with gastrointestinal dysfunction, has been theorized to be initiated within the gut and follow a prion-like spread of pathology through the vagus nerve into the brain [78]. The effects of microbiome-driven inflammation on Parkinson’s pathology were assessed by orally administering bacterial lipopolysaccharide, which caused a rapid increase in alpha synuclein expression in the myenteric neurons of the mouse gut [79]. In humans, Parkinson’s disease is associated with alterations in the microbiota, particularly as regards to *Prevotella* and Enterobacteria [80].

Finally, an interesting preprint article has demonstrated that the microbiome may play a role in the formation of beta-amyloid plaques in the mouse brain. GF Alzheimer’s transgenic mice demonstrated significantly lower levels of beta-amyloid in the brain than conventionally raised transgenic mice. Moreover, the fecal 16S RNA analysis showed that Alzheimer’s transgenic mice had a significantly different microbiome to wild type mice and fecal transplants from transgenic mice, but not wild type mice was able to significantly upregulate the beta-amyloid in the brains of germ-free Alzheimer’s transgenic mice [81].

The assessment of the microbiota in patients with neurodegenerative illnesses is ongoing.

#### The blood-brain barrier

The AHR is widely expressed in the CNS [82, 83]. However, our understanding of the role of the AHR in neurons and supporting cells is still very limited. The BBB is vitally important in the maintenance of CNS homeostasis, and its weakening has been suggested to contribute to neurodegenerative pathology. Breakdown of the BBB at the hippocampus has been correlated with cognitive impairment in humans [84]. Previously, our group reported that the BBB exhibits increased permeability in adult GF mice [57]. Monocolonization with *Clostridium tyrobutyricum* or *Bacteroides thetaiotaomicron* and treatment with sodium butyrate had rescuing effects on BBB permeability and tight junction protein expression [57]. One mechanism of the microbiome-mediated effects on BBB permeability appeared to be related to changes in the expression of tight junction proteins, such as occludin and claudin-5 [57]. A recent report demonstrated that induction of dysbiosis with a mixture of antibiotics caused alterations in the mRNA expression of tight junction proteins in the brain [85], validating, at an mRNA level, the results produced by Braniste et al. in a separate model of microbiome disruption.

The presence of the AHR and expression of its target genes have been shown to be significantly elevated in the microvessels of the brain [83, 86]. Contradictory results have been reported. Via activation by TCDD, the AHR decreases the permeability of the BBB in vivo [87, 88], but increased BBB permeability was observed following exposure to 3-methylcholanthrene [89]. Interestingly, though the increased BBB permeability reported by Braniste et al. has not been assessed in the context of the AHR, a recent study in keratinocytes demonstrated that ligand activation of the AHR elevates occludin and claudin 1 and 4 [90], indicating that a similar AHR-mediated effect could occur in the BBB. One of the most abundant gap junction proteins in the BBB is connexin 43. Connexin 43 expression and gap junction integrity has been shown to be downregulated by AHR activation [91, 92]. The deletion of connexin 43 is known to weaken the BBB, allowing it to open under increased vascular hydrostatic pressure or shear stress [93]. A recent report suggested that connexin 43 is integral to brain immune quiescence [94] and, irrespective of BBB integrity, the deletion of connexin 43 was associated with increased immune cell recruitment across the BBB. Moreover, deletion of connexin 43 leads to activation of the endothelium and chemoattraction, thereby linking a key molecule in the maintenance of BBB integrity with the neuroinflammatory response [94].

#### Neuroinflammation

The role of the AHR in the immune system is being increasingly appreciated [95], and the role of neuroinflammation in psychiatric diseases is also being recognized [96]. One of the hallmarks of neuroinflammation that potentially impacts the neuropsychiatric phenotype [97] and neurodegenerative pathology [98] is the chronic activation of microglia. GF mice have immature microglia with unusual activation properties [99]. Furthermore, microglia from GF mice have altered gene expression profiles similar to the SOD1 mouse model of amyotrophic lateral sclerosis [100]. Although some of the GF microglial phenotypes could be rescued by short chain fatty acid supplementation [99], this does not preclude the possibility of microbiotic interactions through alternate pathways, including the AHR.

AHR mediates both pro-inflammatory and anti-inflammatory effects in microglia [101]. Lee et al. found that AHR activation with FICZ and 3-methylcholanthrene attenuates microglial immune responses. They also demonstrated that silencing the AHR gene with siRNA reduces microglial activation, demonstrating a pro-inflammatory effect of the AHR [101]. Other groups have found similar pro-inflammatory and anti-inflammatory effects of the AHR. Within the CNS of AHR-null mice, microglia accumulate in the retina in a model of age-related macular degeneration [102].

Dietary and microbiotic metabolites, particularly tryptophan metabolites [12], may play an important anti-inflammatory role in the CNS. FICZ was recently shown to modulate astrocyte activity and CNS inflammation through the AHR [103], thereby linking the microbiota directly to neuroinflammatory mechanisms through the AHR. Astrocytes are the most abundant glial cell population in the CNS, participating in metabolism, neuronal transmission, and inflammation [104, 105]. In a mouse model of CNS autoimmunity, CNS inflammation induced a type 1 interferon-mediated response in astrocytes, which induced AHR activation [103]. This AHR response was shown to limit astrocyte inflammation and was increasingly efficacious when mice were supplied dietary tryptophan. To demonstrate that the effects were due to microbiota-mediated metabolism of tryptophan, ampicillin (a broad spectrum antibiotic) was given, which interfered with the effects of dietary tryptophan, and the treatment of mice directly with indoxyl-3-sulfate (a microbial metabolite of tryptophan) led to AHR-mediated anti-inflammatory effects [103]. The dietary metabolite and AHR ligand indirubin-3′-oxime were also shown to inhibit the inflammatory activation of microglia in the rat brain [106]. Whether the immune system regulatory systems exhibited by the AHR in the periphery are relevant to neuroinflammatory responses is not clear. Moreover, as different ligands have different effects on the transcriptional effects of the AHR, the effects of endogenous and exogenous AHR ligands require deep investigation to further elucidate the varying effects of the receptor in the regulation of neuroinflammation.

The pro-inflammatory and anti-inflammatory effects of the AHR are likely due to the complex interactions the receptor can have with other transcription factors. For example, the pro-inflammatory cytokine TNF-alpha is upregulated when microglia are stimulated by lipopolysaccharide, but this effect is attenuated both when the AHR is activated by FICZ and when the AHR is silenced by siRNA due to the complex interactions between the receptor and NF-κB, which can be modulated by the application of AHR ligands [101]. Similarly, Rothhammer et al. found that the AHR-mediated anti-inflammatory effects in astrocytes are due to the limitation of NF-κB activation [103].

#### Ischemic stroke

One interesting neurodegenerative process clearly regulated by the microbiota and microbiota-metabolized AHR ligands is ischemic neurodegeneration. In three separate mouse models of microbiota disruption, the microbiota was shown to impact the outcome of ischemic stroke [107–109]. Depletion of the microbiota with a cocktail of antibiotics decreased survival in a middle cerebral artery occlusion (MCAO) model of murine stroke and severe colitis in mice after stroke. Interestingly, that study found no significant difference in infarct size 1 day after stroke [107]. In a separate model in which the microbiota of mice was altered, not depleted, with amoxicillin and clavulanic acid, infarct volume was significantly reduced compared to mice with a healthy microbiota [108]. This microbiota-stroke effect may be bidirectional, as Singh et al. demonstrated that particularly large infarcts can cause dysbiosis within the microbiota, possibly potentiating neuroinflammatory effects within the CNS [109]. Benakis et al. demonstrated that intestinal IL-17^+^ γδ T cells, which were reduced in their model of dysbiosis, accumulate in the meninges after stroke and are responsible for a neuroinflammatory response that potentiates damage after ischemic insult [108]. Interestingly, the AHR alters the function of γδ T cells and its stimulation with FICZ elevates IL-17 production in these cells [110]. In human illness, elevated serum levels of KYNA during the acute phase of stroke have been correlated with worse neuropsychiatric outcomes in stroke patients [111]. Similarly, IDO activity, as determined by the KYN to tryptophan ratio, is positively associated with stroke severity [112], and other elements of the KYN metabolic pathway have been correlated with infarct size in stroke [113]. To evaluate the specific role of the AHR in stroke, Cuartero et al. used the MCAO stroke model to demonstrate that the receptor is upregulated and activated after ischemic insult [114]. Pharmacological inhibition of the AHR resulted in a smaller infarct size and greater functional outcomes, and stimulation of the AHR resulted in increased infarct volume [114]. KYN levels in the brain were also elevated in this model of stroke and, through activation of the AHR, played a deleterious role in cerebral ischemia [114]. How the microbiota interacts with tryptophan metabolism to affect the AHR in neural ischemia is still unknown.

## Summary and further directions

**Fig. 3:**
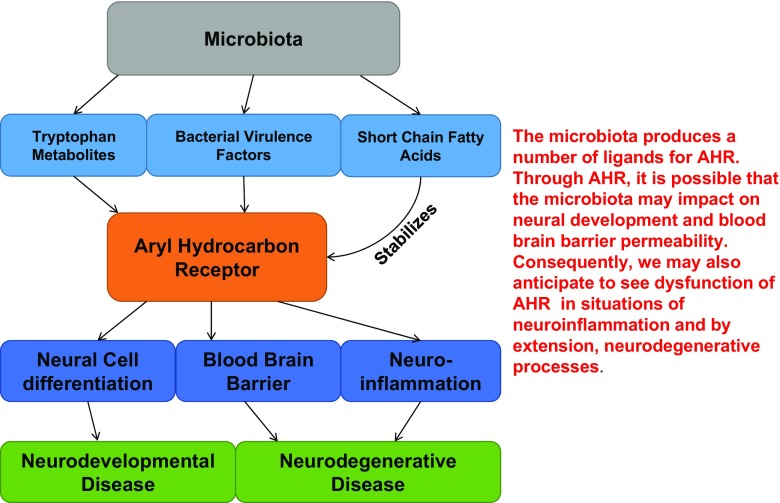
Proposed model. The activities of the microbiota through the aryl hydrocarbon receptor (*AHR*) on the central nervous system

As mounting evidence supports the holobiont model of the host and its microbiome, one of the most important questions that the researchers are facing is the mechanisms by which the microbiota communicates with the host. The AHR is an evolutionarily conserved ligand-induced receptor involved in host-environment interactions. Despite its well-known responsiveness to man-made compounds, such as TCDD, in invertebrates, the AHR does not elicit a response to dioxin. Therefore, AHR must execute other evolutionarily important roles in development and homeostasis. Interestingly, we and others have found that AHR responds to microbiome-mediated ligands engaging host immune and metabolic responses. Moreover, many AHR ligands cross the BBB, implying a role of AHR in the CNS (Fig. 3). While preparing this review, we have realized that there are much more to be learned about the AHR signaling pathway and its impact on CNS development and function. The pleiotropic action of AHR and its wide expression pattern may also hold hope for the development of new microbiome derived compounds that support the metabolic homeostasis within the holobiont.
